# NoxO1 regulates EGFR signaling by its interaction with Erbin

**DOI:** 10.1016/j.redox.2024.103396

**Published:** 2024-10-16

**Authors:** Dana Maureen Hebchen, Tim Schader, Manuela Spaeth, Niklas Müller, Johannes Graumann, Katrin Schröder

**Affiliations:** a Institute for Cardiovascular Physiology, Goethe University Frankfurt, Germany; b German Center of Cardiovascular Research (DZHK), Partner site RheinMain, Frankfurt, Germany; c Biomolecular Mass Spectrometry, Max Planck Institute for Heart and Lung Research, Bad Nauheim, Germany; d Institute of Translational Proteomics, Biochemical/Pharmacological Centre, Philipps-Universität Marburg, Germany

**Keywords:** NADPH oxidase, EGF signaling, Erbin, NoxO1

## Abstract

NADPH oxidase organizer 1 (NoxO1) is a scaffold cytoplasmic subunit of the reactive oxygen species (ROS) forming Nox1 complex and involved in angiogenesis, differentiation, and atherosclerosis.

We found that overexpression of NoxO1 without simultaneous overexpression of any other component of the active Nox1 complex inhibited EGF-induced wound closure and signaling, while NoxO1 KO yielded the opposite effect. Accordingly, we hypothesize NoxO1 to exert Nox1 independent functions.

Using the *BioID* technique, we identified ErbB2 interacting protein (Erbin) as novel interaction partner of NoxO1. Colocalization of NoxO1 with EGFR, as well as with Erbin validated this finding. EGF treatment interrupted colocalization of NoxO1 and EGFR. EGF mediated kinase activation was delayed in NoxO1 overexpressing cells, while knockout of NoxO1 had the opposite effect.

In conclusion, Erbin was identified as a novel NoxO1 interacting protein. Through the subsequent interaction of NoxO1 and EGFR, NoxO1 interferes with EGF signaling. The results of this study suggest a potential role of NoxO1 as an adaptor protein with functions beyond the well-established enabling of Nox1 mediated ROS formation.

## Graphical abstract

**Figure:**
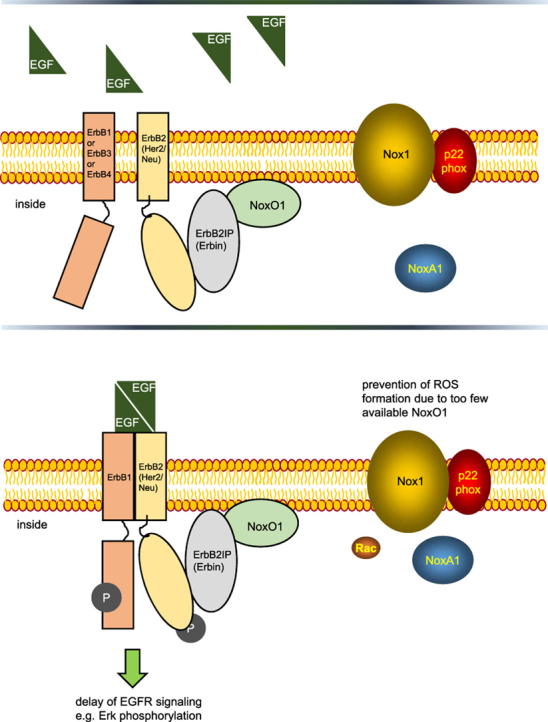

## Teaser

Significance statement, Here we provide evidence for a new function of NoxO1, which is the organizing cytosolic subunits of the multi-subunit enzyme Nox1. We discovered an interaction of NoxO1 and Erbin and thereby a role in EGF signaling.

## 1 Introduction

NADPH oxidases (Nox) are responsible for the production of reactive oxygen species (ROS) [1,2]. ROS play a role in a number of physiological processes, including host defense and vascular function [3,4]. NADPH oxidase organizer 1 (NoxO1) is a cytosolic subunit of the Nox1-centered NADPH oxidase [5]. NoxO1 is highly expressed in the epithelia of the intestine, pancreas, and lung (The Human Protein Atlas ENSG00000196408). NoxO1 serves as an organizer for the components of the Nox1 complex. Notwithstanding its well-documented capacity to facilitate a constitutive superoxide formation by the Nox1 complex, NoxO1 is susceptible to phosphorylation and ubiquitination [6]. Both modifications have been demonstrated to reduce superoxide formation derived from the Nox1 complex [7]. Indeed, the availability of NoxO1 directly controls the superoxide-forming activity of Nox1 [8].

Redox-sensitive effectors are present in numerous signaling networks, including those downstream of growth factors [9]. One such growth factor is epidermal growth factor (EGF). The epidermal growth factor receptor (EGFR) and its family members, the HER/ErbB receptors, are transmembrane tyrosine kinases that regulate fundamental cellular processes, including proliferation, survival, and migration [10,11]. The EGFR family comprises four members (ErbB1-4) that bind to 14 ligands, forming homo- or heterodimers upon ligand binding, which then undergo auto-phosphorylation of the cytoplasmic kinase domains. The orphan receptor ErbB2 (Her2), which exhibits the highest catalytic activity, is the preferred dimerization partner of ErbB1 (EGFR). ErbB2 is a prominent oncogene with high importance in breast cancer [12].

Many effects induced by EGF are opposed by NoxO1 [13]. While EFG has been demonstrated to promote proliferation and angiogenesis [14], both processes are inhibited by NoxO1 [7]. In contrast, NoxO1 has been demonstrated to mediate apoptosis [15], while EGF has been shown to elicit opposing effects [16]. It is not possible to securely relate all of the aforementioned effects to ROS formation or even the use of ROS as second messengers. Additionally, NoxO1 expression frequently exceeds that of other subunits, such as NoxA1 and Nox1, in numerous cell types [8]. Consequently, alternative possibilities must be considered.

In this study, we employed the proximity-dependent Biotin Identification (BioID) technique and identified Erbin as a novel interaction partner of NoxO1, which enables direct modification of EGF- and EGFR-mediated signal transduction by NoxO1.

## 2 Results

### 2.1 NoxO1 accelerates EGF-induced wound closure

**Fig. 1:**
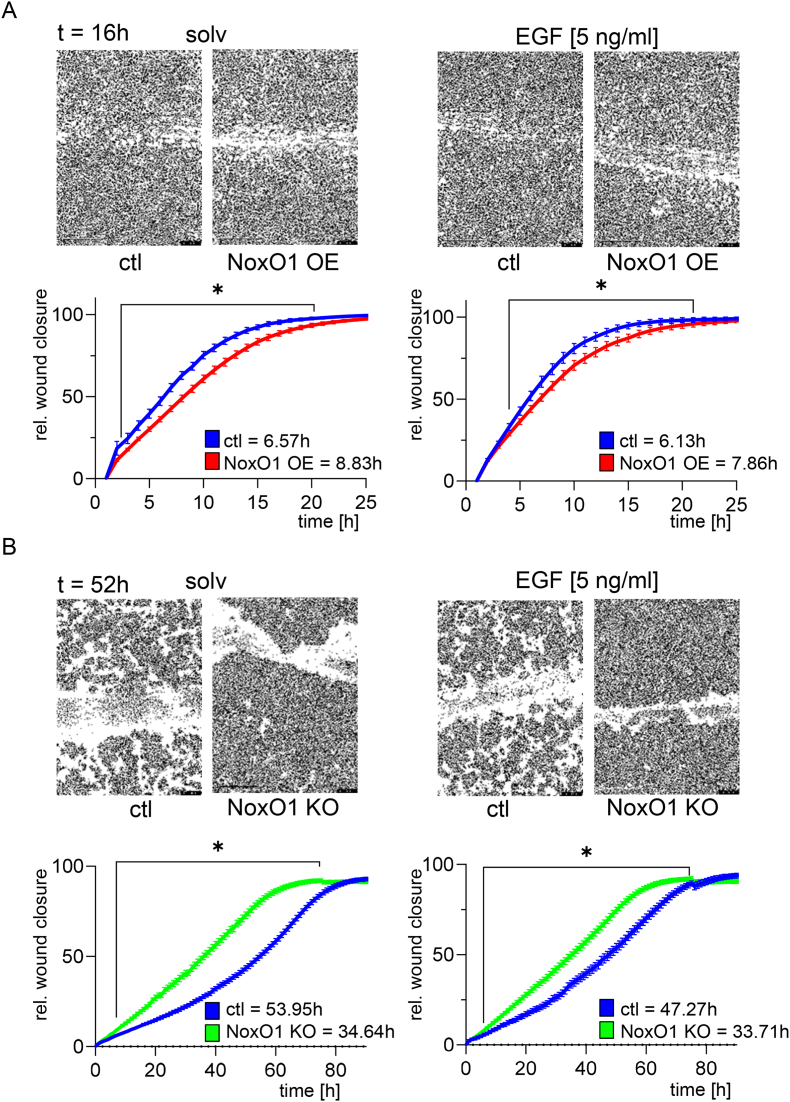
**NoxO1 decelerates wound closure.** Representative images and statistical analysis of Scratch-Wound assays with (A) Hek293 cells overexpressing NoxO1 (OE) or empty vector (ctl) and (B) MCF7 cells with NoxO1 KO (KO) or not (ctl); time for 50 % wound closure is indicated in the graph; n = 4; ∗p < 0.05 in Two-Way ANOVA + Tukey post hoc test; mean ± SEM.

In a previous study, we observed that NoxO1 was moderately expressed in Hek293 cells, whereas its expression in MCF7 breast cancer cells was markedly elevated. Accordingly, we employed Hek293 cells as a model for overexpression of NoxO1 and MCF7 cells as a model for NoxO1 knockout. The overexpression of NoxO1 in Hek293 cells resulted in a delay in wound closure in a scratch-wound assay, as illustrated in Fig. 1A. Conversely, the knockout of NoxO1 in MCF7 cells resulted in accelerated wound closure (Fig. 1B). The time required for wound closure varied significantly depending on the cell type, with a duration of 25 h observed in Hek293 cells and up to 80 h in MCF7 cells. However, the results suggest that NoxO1 exerts negative regulation in basal and EGF-induced wound closure.

### 2.2 EGF induced ROS-formation is enhanced by NoxO1 overexpression

**Fig. 2:**
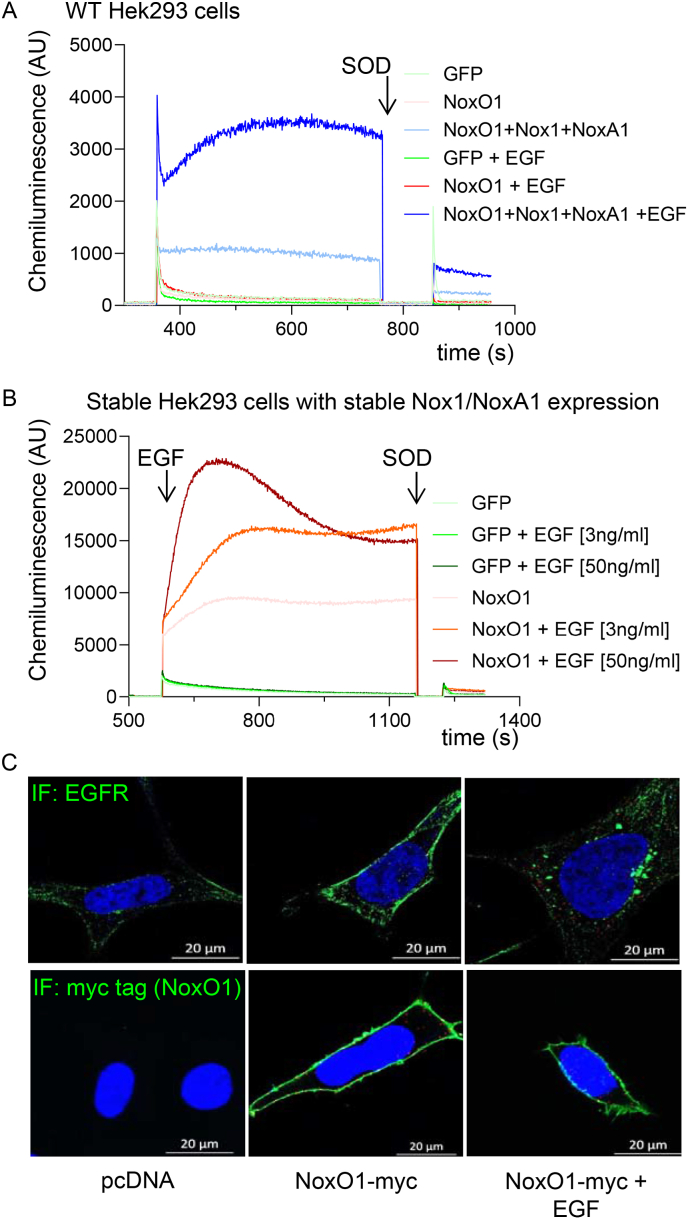
**EGF induces acute ROS formation, mediated by a Nox1 centered NADPH oxidase.** Superoxide production (L-012) measurement in (A) Hek293 cells overexpressing only NoxO1 or all components of the Nox1 centered NADPH oxidase (Nox1+NoxA1+NoxO1) treated with EGF (50 ng/ml) 15 min before the actual measure. (B) Hek293 with constitutive overexpression of Nox1 and NoxA1 and transient expression of NoxO1 with acute addition of EGF to the cells in the measure chamber; Representative measurement traces; (C) Immunofluorescence of endogenous EGFR and overexpressed myc-taged NoxO1 in Hek293 cells.

Although it is established that NoxO1 plays a role in the constitutive production of ROS by the Nox1-centered NADPH oxidase [17], our findings indicate that EGF stimulation enhances ROS formation by the NoxA1/NoxO1/Nox1 complex (Fig. 2). The induction of superoxide formation was observed in both experimental conditions: long-term (Fig. 2A) and acute activation within seconds after EGF treatment (Fig. 2B). EGF treatment has no effect on NoxO1 plasma membrane localization (Fig. 2C). It has been demonstrated that NoxO1 can be phosphorylated in response to ligand stimulation. However, this has yet to be shown to contribute to the acute activation of ROS formation mediated by the Nox1 complex [18]. Instead, it has been observed that increased levels of available NoxO1 act as an activator of Nox1-mediated ROS formation in a dose-dependent manner [8]. EGF-induced ROS formation reached similar levels in both cases, indicating saturation of its receptors [19]. It can be concluded that upon stimulation of the cells with EGF, NoxO1 is released from the Erbin/EGFR complex, which may represent an intracellular pool. NoxO1 then migrates to Nox1, and assembles the entire complex, which eventually enables Nox1-mediated ROS formation. Accordingly, the objective was to identify potential interaction partners of NoxO1 that may retain it in unstimulated cells and release it upon stimulation, for example, with EGF.

### 2.3 Erbin as novel interaction partner of NoxO1

**Fig. 3:**
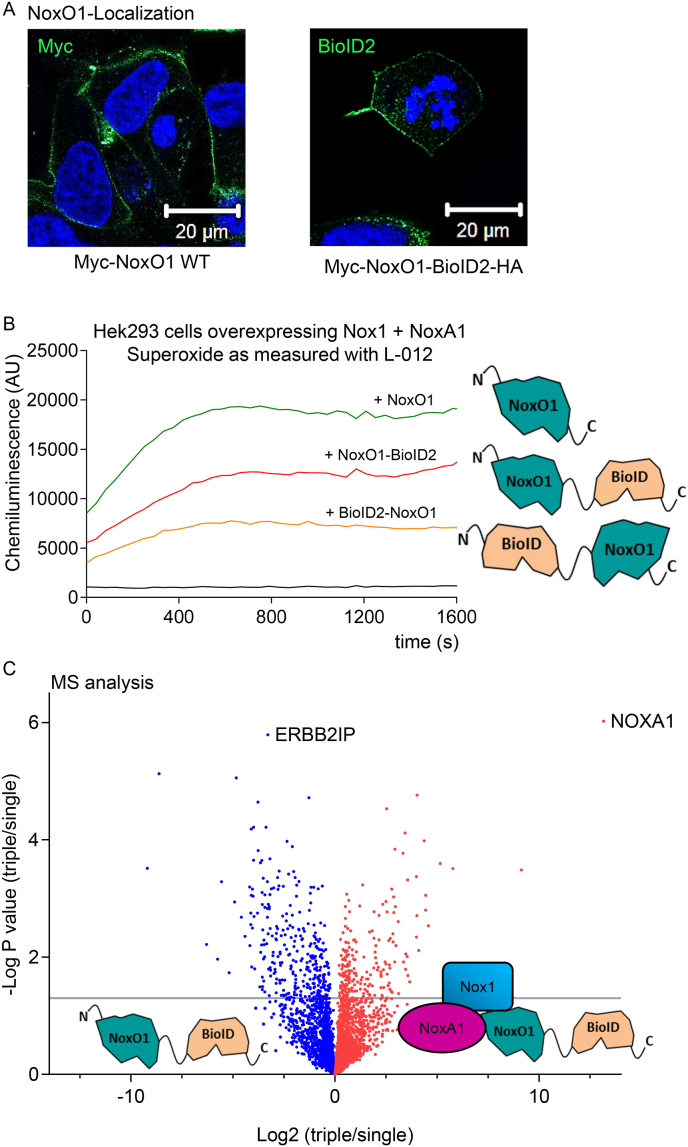
**NoxO1, if not together with the Nox1 centered NADPH oxidase interacts with ErbB2IP (Erbin).** (A) immunofluorescence of NoxO1 constructs as indicated (B) Superoxide measurement (L-012) in Hek293 cells overexpressing the NoxO1 construct indicated; (C) Identified biotinylated proteins in Hek293 cells transfected with the NoxO1-BioID construct in the absence or presents of Nox1 and NoxA1.

To that end, we employed the BioID technique, as previously described [20,21]. The NoxO1-BioID2 fusion constructs were evaluated for expression, membrane translocation, and their capacity to induce ROS formation as part of the Nox1 complex (Fig. 3 A&B). A mass spectrometry-based approach revealed that NoxA1 is the most probable interacting protein with NoxO1 when all components of the No1-centered NADPH oxidase (Nox1, NoxA1, NoxO1) are overexpressed (Fig. 3C). Upon overexpression of NoxO1, Erbin was identified as a top target of biotinylation.

### 2.4 Localization and endocytosis of EGFR, Erbin and NoxO1

**Fig. 4:**
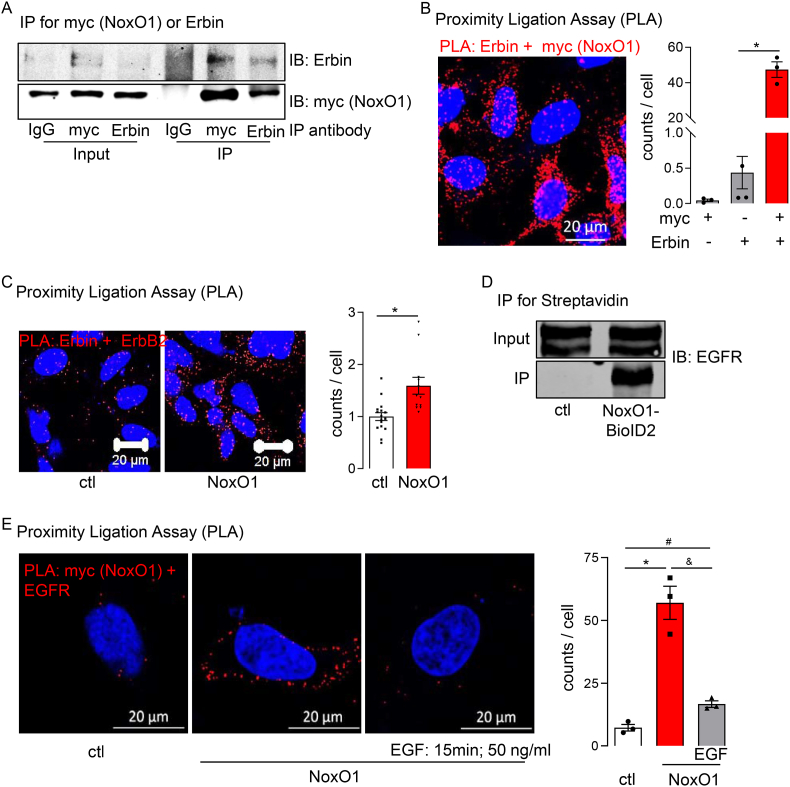
**EGF interrupts Erbin mediated interaction of NoxO1 and EGFR.** (A) Immunoprecipitation for streptavidin (biotin) from Hek293 cells overexpressing empty vector (ctl) or NoxO1-BioID2 as indicated with detection of Erbin and myc (NoxO1); (B) Proximity ligation assay for myc (NoxO1) and Erbin in Hek293 cells overexpressing NoxO1; (C) Proximity ligation assay in Hek293 cells overexpressing empty vector (ctl) or NoxO1, detection of Erbin and ErbB2; (D) Immunoprecipitation for streptavidin (biotin) from Hek293 cells overexpressing empty vector (ctl) or NoxO1-BioID2 as indicated with detection of EGFR; (E) Proximity ligation assay in Hek293 cells overexpressing empty vector (ctl) or NoxO1 and treated without or with EGF as indicated, detection of myc (NoxO1) and EGFR; n = 3; ∗p < 0.05 ctl vs. NoxO1, #p < 0.05 ctl vs. NoxO1 + EGF, & p < 0.05 NoxO1 vs. NoxO1 + EGF in Two-Way ANOVA + Tukey post hoc test; mean ± SEM.

The proposed interaction between NoxO1 and Erbin was verified by using proximity ligation assays, co-immunoprecipitation of NoxO1 and Erbin, and Western blot analyses of NoxO1-BioID biotinylated proteins (Fig. 4 A&B). Given that ErbB2 is the preferred dimerization partner for EGFR, it was anticipated that Erbin and, consequently, NoxO1 would colocalize with EGFR. Proximity ligation assays and co-immunoprecipitation experiments demonstrated a direct interaction between NoxO1 and EGFR (Fig. 4C–E). Following EGF stimulation, NoxO1 was observed to dissociate from EGFR. As a result, NoxO1 would be able to translocate to the Nox1 complex, leading to ROS formation. Indeed, overexpression of Erbin and thereby reducing the availability of NoxO1 reduced EGF-induced ROS formation, whereas Erbin knockout resulted in an increase in ROS formation without any further stimulus (Supplemental Fig. 1).

It can be concluded that EGFR, ErbB2, Erbin, and NoxO1 form a complex at the cell membrane. It is yet to be determined whether the dissociation of NoxO1 and EGFR is a prerequisite for EGFR signaling.

### 2.5 Erbin and NoxO1 inhibit EGF signaling

**Fig. 5:**
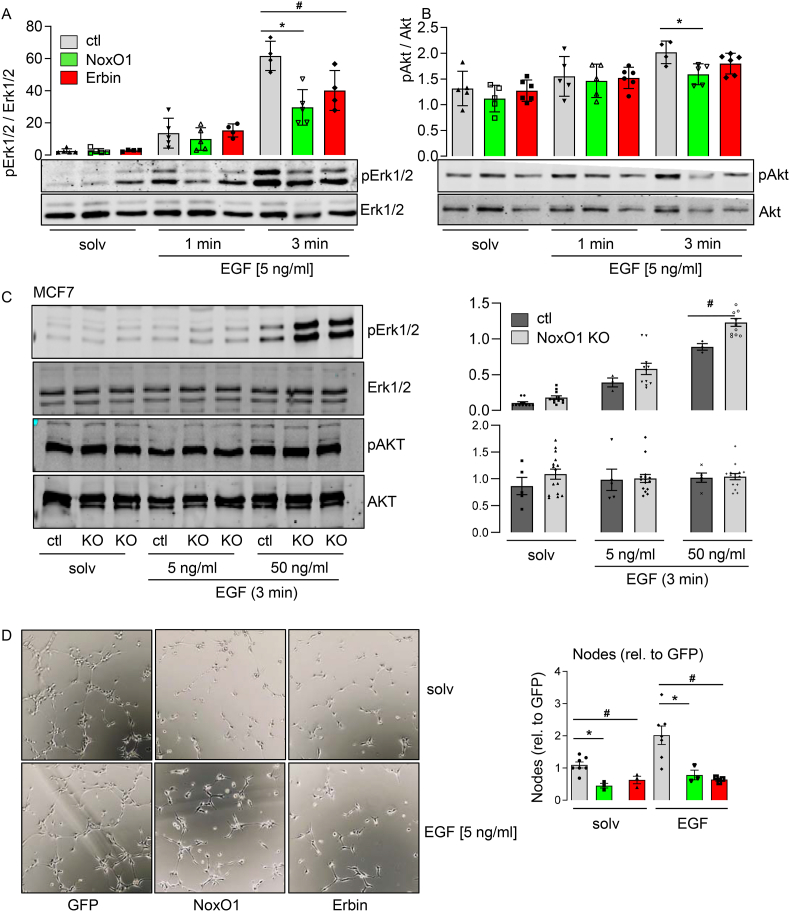
**NoxO1 and Erbin overexpression reduce EGF induced signaltransduction.** (A & B) Western blot for phosphorylated and total (A) Erk1/2 and Akt (B) in Hek293 cells overexpressing empty vector (ctl), NoxO1 or Erbin (C) MCF7 without (ctl) and with NoxO1 knock out (KO) treated without or with EGF as indicated; (D) Tube formation assay in HUVECs overexpressing empty vector (ctl), NoxO1 or Erbin treated without or with EGF over night; n = 3–5; ∗p < 0.05 ctl vs. NoxO1, #p < 0.05 ctl vs. Erbin in Two-Way ANOVA + Tukey post hoc test; mean ± SEM.

We conducted an analysis of the EGF-induced activation of EGFR and the subsequent phosphorylation of downstream kinases, with and without the overexpression of Erbin and NoxO1. Following brief EGF stimulation in Hek293 cells, both NoxO1 and Erbin were observed to inhibit Erk1/2 and Akt phosphorylation (Fig. 5A and B). Conversely, the depletion of NoxO1 in MCF7 cells resulted in an increase in EGF-induced Erk1/2 phosphorylation (Fig. 5C). Subsequently, Human Umbelical Vein Endothelial Cells (HUVECs) were employed as an additional model to corroborate the observations made. Overexpression of either NoxO1 or Erbin diminished both basal and EGF-induced tube formation (Fig. 5D). The data substantiate the hypothesis that NoxO1 serves as a novel regulator of EGFR signaling.

## 3 Discussion

In this study, we found Erbin as a new interaction partner of NoxO1. NoxO1 contains several domains that are feasible for protein-protein interactions like a Proline Rich Region (PRR) and two SH3 domains [22]. The cytoplasmic scaffold ErbB Interacting Protein (Erbin) localizes to basolateral membranes where it establishes cell polarity [23]. Erbin can undergo phosphorylation of unknown function. Interestingly, Erbin interferes with many signaling pathways by interacting with Rho G proteins, the p120 catenin family, Wnt proteins etc. [24,25]. Moreover, Erbin has been implicated with Transforming Growth factor β signaling on a special subset of endosomes [26,27]. The role of Erbin as tumor suppressor and its potential as prognostic marker are still under debate [28].

As Erbin specifically binds ErbB2 [11], Erbin and NoxO1 can only act jointly, when the EGFR consists of ErbB1 and ErbB2, which in fact is the preferred condition in many cases [29]. Importantly, ErbB2 (also known as Her2) is a prominent oncogene and ErbB1/ErbB2 dysregulation or amplification is associated with poor prognosis in several cancer types [12,30]. Recently, a ligand-independent ErbB2 activity has been proposed to be sufficient for preventing apoptosis in Madin-Darby canine kidney cells [29].

The data provided in this study indicate that NoxO1 induces overall changes in EGF signaling, which may largely depend on its interaction with Erbin. From the results in this study, the following scenario is possible: Without a stimulus, Erbin and NoxO1 associate and little or no ROS is formed. In fact, NoxO1 appears to intensify the association of EGFR and Erbin. By that mechanism, NoxO1 delays subsequent signaling after EGF bound the EGFR. Eventually, once the complex of EGFR, Erbin and NoxO1 dissociates, EGF induced signaling starts to develop and MAPKinases such as Erk are phosphorylated. The released portion of NoxO1 stays at the membrane and migrates towards Nox1, where it eventually enables ROS formation. The observed delay in EGF induced MAPKinase phosphorylation in NoxO1 and Erbin overexpressing cells may underline its role as an antagonist for EGF induced proliferation and survival. EGFR/Her2 dysregulation or amplification is associated with poor prognosis in several cancer types [30]. Overcoming therapeutic resistance of anti-EGFR therapies is a challenge that demands novel molecular targets and a deeper understanding of aberrant EGFR trafficking [[31], [32], [33]]. Therefore, we suggest that targeting NoxO1 instead of EGFR may represent an attractive option for specific alteration of ErbB2 dependent EGF signaling.

### 3.1 Limitation

A significant discovery of the present study is that NoxO1 plays a regulatory role in cellular signaling, interacts with a diverse range of proteins beyond those belonging to the NADPH oxidase family, and exerts effects independently of ROS. EGF induces ROS formation by the Nox1-centered NADPH oxidase. It is possible that EGF induces NoxO1 phosphorylation. However, no evidence was found to suggest that EGF treatment results in a stronger association between Nox1 and NoxO1. Further experiments are required to address these questions, and the development of reliable antibodies would facilitate this.

## 4 Materials and methods

If not stated otherwise, human genes and proteins are addressed.

### 4.1 Cell lines and cell culture

Human cell lines (Hek293, MCF7) were purchased from ATTC (Manassas, USA). All cells were cultivated in Minimal Essential Medium (MEM, #11095080 Gibco) with 1 mM Sodium pyruvate (#M7145 sigma), 0.1 mM Non-essential Amino acids (#S8636,sigma), 0.5 % Penicillin-Streptomycin (#15140-122 sigma) and 8–20 % fetal calve serum (FCS, #f7524 sigma). For MCF7 cells, 0.01 mg/ml human insulin (#I9278, sigma) was supplemented. Cells were cultured under 5 % carbon dioxide atmosphere at 37 °C. Prior to stimulations with Epidermal Growth Factor (EGF, #AF-100-15, Peprotech) or other treatments, cells were serum-starved for 6h in MEM. Human umbilical vein endothelial cells (HUVECs) were obtained from Lonza (#CC-2519, Lot No. 371074, 369146, 314457, 192485, 186864, 171772, Walkersville, USA) and PeloBiotech (#PB-CH-190-813, Lot No. QC-18P13F11, Planegg, Germany). HUVECs were cultured in dishes coated with gelatin in endothelial growth medium (EGM). EGM was composed of endothelial basal medium (EBM) supplemented with human recombinant epidermal growth factor (EGF), EndoCGS-Heparin (PeloBiotech), 8 % FCS, penicillin (50 U/ml) and streptomycin (50 μg/ml) (#15140–122, Gibco).

### 4.2 Tube formation assay

Tube formation assays were performed in μ-Slide Angiogenesis coverslips (#81507, Ibidi, Planegg, Germany). Matrigel was thawed on ice overnight. 10 μl of matrigel (#356231, Corning, Corning, USA) were added per well and allowed to polymerize for 30 min at 37 °C. HUVECs were transfected and after 24 h cells were starved in EBM+0.1 % BSA. Cells were, trypsinized and counted. 5000 cells were seeded onto the matrigel in 50 μl EBM with 1 % FCS. Tube formation was analyzed by counting nodes using ImageJ.

### 4.3 Overexpression systems

For transient overexpression, transfection was carried out with 1 μg/ml polyethyleneimine (PEI, #408727 sigma) or the Lipofectamine3000® Kit (#L3000001 Invitrogen) for 4–6 h at 37 °C in MEM without supplements. After exchange of MEM to growth media (see section cell lines), overexpression was allowed for 1 day before performing experiments.

Constitutive overexpression was generated by lentiviral transduction followed by selection with 400 μg/ml Hygromycin (#ALX-380-309-G001 Enzo) or 2 μg/ml Puromycin (#0240.4 Carl Roth). Lentiviral particles were produced in Lenti-X™ 293T cells (purchased from Takara) by transfection with 1 μg/ml PEI together with the packaging plasmids psPAX2/pmD2.G (#12260, #12259 Addgene) and the encoding plasmid. After 1–2 days, lentiviral particles were harvested from the supernatant and tested with Lenti-X™ GoStix™ Plus (#631280 Takara). Host cells were infected with 1 ml supernatant and 8 μg/mL Polybrene (#TR-1003-G Merck) for 1 day. Selection was started after 1–2 days. As control, cells were transduced with an empty vector construct (Table 1). All plasmids were verified by Sanger sequencing at Microsynth Seqlab GmbH (Göttingen, Germany).

**Table 1:** Overexpression systems.

protein expressed		backbone	tag for detection
Myc-BioID2-MCS	transient	pcDNA 3.1	myc
BioID2-hNoxO1-HA			HA
hNoxO1-BioID2-HA			HA
Nox1		pCMV.6-entry	c-myc, Flag-DDK
NoxA1			
NoxO1			
eGFP		pEGFP-C1	GFP
Erbin		pCl-neo	c-myc
(empty vector)	constitutive	pLV-EF1a-IRES-Hygro	–
NoxO1			
Nox1+NoxA1		EF1aFull-hOct4-F2A-hKlf4-IRES-hSox2-P2A-hcMyc-W-loxP

**Table 2:** Primary antibodies.

Target	host	manufacturer	Product Reference
AKT	mouse	Cell Signaling	#2920
	mouse	BD	#610457
EGFR	rabbit	Invitrogen	#PA 1–1110, #PA5-85476
Erbin	rabbit	Thermo Fisher	#PA566288
Erk	mouse	Cell Signaling	#4696
HA-tag	rabbit	Cell Signaling	#3724S
IgG	mouse	Diagenode	#C15410206
myc-tag	goat	Bethyl/Biomol	#A190-104A
NoxO1	rabbit	Eurogentec	#2110891 (customized)
pAkt (Ser473)	rabbit	Cell Signaling	#40585
pEGFR (Tyr1068)	mouse	Thermo Fisher	#MA515199
pEGFR (Tyr11101)	mouse	Abcam	#ab76195
pErbin (Tyr1104)	rabbit	Thermo Fisher	#PA5-103132
pErk1/2 (Thr202/Tyr204)	rabbit	Cell Signaling	#9101

### 4.4 Gene knockout by CRISPR/CAS system

*NoxO1* and *Erbin* were knocked out by applying the lentiviral CRISPR/CAS technique in Hek293 cells. Guide RNAs (gRNAs) were designed at the crispor.tefor.net platform (Table 4) and cloned into lenti CRISPRv2 (#52961 addgene) backbone through Golden Gate Assembly [60]. Briefly, sense and antisense gRNAs were annealed at 98 °C for 5 min and subjected to restriction and ligation in a thermocycler. PCR product was transformed into the *E.coli* DH5a strain and positive clones selected by Ampicillin resistance. Plasmids were isolated using the GeneJET Plasmid Mini Kit and sequenced at MicroSynth Seqlab GmbH (Göttingen, Germany). Production of lentiviral vectors and transduction were conducted like for overexpression systems. Gene knockout was verified by PCR, Western blot and ROS formation.

**Table 3:** Secondary antibodies.

Target	label	host	manufacturer	Product Reference
Anti-biotin Streptavidin IRDye®	680RD	–	LI-COR	#926-68079
	800CW			#926-32230
Anti-goat	AF488	donkey	Invitrogen	#A11055
Anti-mouse	AF488	donkey	Invitrogen	#A21202
	AF546			#A10036
	AF647			#A31571
Anti-mouse IRDye®	680RD	donkey	LI-COR	#926-68072
	800CW			#926-32212
Anti-rabbit	AF488	donkey	Invitrogen	#A21206
	AF546			#A10040
	AF647			#A31573
Anti-rabbit IRDye®	680RD	donkey	LI-COR	#926-68073
	800CW			#926-32213

**Table 4:** gRNAs for CRISPR/Cas9 gene knockout.

gene	sense (5′-3′)	antisense (5′-3′)
***Erbin***	CACCGTTACAGCAGTTGCCCCCAG	AAACCTGGGGGCAACTGCTGTAAC
***NoxO1***	CACCGAAGCCGCCACCGCGGCATCAGGG	AAACCCCTGATGCCGCGGTGGCGGCTTC
	AAACCCCTGATGCCGCGGTGGCGGCTTC	CACCGCGCGGTCAGATCTCCGCAGCAGG
	CACCGCGCGGTCAGATCTCCGCAGCAGG	AAACCCTGCTGCGGAGATCTGACCGCGC
	AAACCCTGCTGCGGAGATCTGACCGCGC	CACCGCACTGAAACTGGGTATCGGGGG
	CACCGCACTGAAACTGGGTATCGGGGG	AAACCCCCCGATACCCAGTTTCAGTGC
	AAACCCCCCGATACCCAGTTTCAGTGC	CACCGCCAGTGGGAGGCAGCCGCGTGGG
	CACCGCCAGTGGGAGGCAGCCGCGTGGG	AAACCCCACGCGGCTGCCTCCCACTGGC
	AAACCCCACGCGGCTGCCTCCCACTGGC	CACCGGTCCCTCACCCGGATGGCAGGG
	CACCGGTCCCTCACCCGGATGGCAGGG	AAACCCCTGCCATCCGGGTGAGGGACC

### 4.5 Genomic DNA isolation and PCR

Confluent cells were mechanically detached from a 24-well dish and incubated for 30 min at 56 °C at 800 rpm in warm lysis buffer (5 mM Tris-HCl pH 8.5 #AE15.3 Carl Roth, 10 mM NaCl #31434-5 KG-R sigma 0.2 % SDS #CN30.3 Carl Roth, 5 mM EDTA #ED-1KG sigma, 3 μg Proteinase K #P2305-25 MG sigma). Lysates were spin down and the supernatant including genomic DNA (gDNA) precipitated with isopropanol. After pelletizing and several washings with 70 % ethanol, gDNA was dried and resuspended in water. 100–300 ng gDNA were used for PCR with primers flanking the CRISPR target side (Table 5). PCR product was separated by electrophoresis in 1.5 % universal agarose (#BS20.46.1009 VWR) gel in Mini Plus Horizontal chambers (Carl Roth). Gels were stained with Roti-Stain® and visualized at the Gel Stick (Intas).

**Table 5:** Primers for CRISPR validation through PCR or sequencing.

gene	forward (5′-3′)	reverse (5′-3′)
***NoxO1***	CCTTGAGCTGCCTGAATTCG	ACCTGGCTGGGTCCTTAGTG
	ACCTGGCTGGGTCCTTAGTG	TCCAGTGGGAGTCACTGATG
	TCCAGTGGGAGTCACTGATG	ACGAATTCAGGCAGCTCAAG
	ACGAATTCAGGCAGCTCAAG	ACCCAGCCAGGTCTTACTTG
	ACCCAGCCAGGTCTTACTTG	CGCCCATTTCAGGAATCTGC
	CGCCCATTTCAGGAATCTGC	CCGAGAAGCTTTGGGAGAAC
***GAPDH***	TGGTGTCAGGTTATG CTGGGCCAG	GTGGGATGGGAGGGTGCTGAACAC
***Erbin***	TGCAGTCAAAGACACTTTGTGG	–

### 4.6 ROS measurement with chemiluminescence

Reactive oxygen species (ROS) measurements assessing superoxide were carried out with L-012 (8-Amino-5-chloro-2,3-dihydro-7-phenyl-Pyrido[3,4-d]pyridazine-1,4-dione).

Living cells were resuspended in HEPES-Tyrode buffer (137 mM NaCl #31434-5 KG-R, 2.7 mM KCl #P9333, 0.5 mM MgCl #M8266, 1.8 mM CaCl2 #C7902, 5 mM d-Glucose #16301, 0.36 mM NaH2PO4∗H2O # 106346, 10 mM HEPES # H-3375, all from sigma) containing 200 μM L-012 (#120–04891, WAKO Chemicals). ROS production of 100 000 cells was assessed by chemiluminescence at 37 °C in a 6-channel luminometer. For quenching of superoxide, 20U superoxide dismutase (#S7571, sigma) was added.

### 4.7 Immunofluorescence and confocal microscopy

Immunofluorescence (IF) was performed on 8-well μ-slides (ibidi). After treatment, cells were fixed with Roti® Histofix (Carl Roth), washed with Dubecco's Phosphate-buffered saline (DBPS, #14040133 Gibco) and 2 % l-glycine (#A1377,5000 AppliChem). Cells were permeabilized with 0.05 % Triton-X 100 (Carl Roth). Unspecific binding sites were blocked with 3 % bovine serum albumin (BSA, #A8412, sigma). Primary antibodies (Table 2) were incubated (1:200) overnight and stained with AlexaFluor-conjugated secondary antibodies (1:500) (Table 3). Nuclei were stained with 0.1 μg/ml DAPI (4′,6-diamidino-2-phenylindole, #D9542 sigma). Slides were stored in the dark until detection with a confocal laser scanning microscope (LSM800, Zeiss).

### 4.8 Proximity Ligation Assay

Proximity Ligation Assay (PLA) to visualize protein-protein interactions was performed using the Duolink® In situ Orange Kit (#DUO92007 sigma) according to the manufacturer's protocol.

Briefly, samples were prepared like for IF with the 2 primary antibodies against the interacting proteins (1:500 each). Secondary antibodies matching the antibody species and carrying oligonucleotides were ligated and amplified in a rolling circle reaction. Nuclei were stained with 0.1 μg/ml DAPI (4′,6-diamidino-2-phenylindole, #D9542 sigma). PLA signals were imaged with a laser scanning microscope (LSM800, Zeiss). Fluorescence was excited at 554 nm. PLA was combined with IF by co-incubation of primary antibodies (1:300) with the IF antibody for a third target. Secondary antibody for IF (1:500) was added after the last PLA polymerization step.

### 4.9 *BioID* technique

BioID technique was used to screen for novel NoxO1 interaction partners as previously described [34,35]. Proximity-based biotinylation was performed with transient overexpression N- or C-terminal NoxO1-BioID2 fusion constructs in Hek293 cells. Overexpression of BioID2 only served as control. NoxO1-BioID2 fusion proteins were validated by immunofluorescence and ROS production. Cells were incubated with 50 μM biotin (#B4639, sigma) in growth media for 24h followed by cell harvest and lysis. Biotinylated proteins were precipitated with streptavidin C1 magnetic beads (Dynabeads™ MyOne™ 65001Thermo Fisher). NoxO1 interaction partners were identified either by mass spectrometry or Western blot (n = 3). Sample reproducibility was validated using PAGE/silver staining. Interactomes were identified by GelC-MS2/label free quantitation at the Max-Planck Institute for Biomolceular Mass Spectrometry in Bad Nauheim. Mass spectrometry data were analyzed with Max Quant (1.6.2.3) and Excel 2016.

Background proteins were determined by BioID2-only samples and excluded from the candidates. Known contaminants and false discovery remnants were removed. Data were log2 transformed and missing values replaced by normal distribution. Significance of interacting proteins was analyzed by student's t-test.

### 4.10 SDS-PAGE and western blot

Cells were lysed in TritonX-100 lysis buffer (250 mM Tris∗HCl pH7.4 #AE15.3 Carl Roth, 750 mM NaCl #S/3160/65 fisher, 50 mM NaPPi #106391 Merck, 100 mM NaF #201154 sigma, 10 % Triton-X #3051.3 Carl Roth, 2 mM Orthovanadate #A2196 AppliChem, 10 mM Okadaic Acid #ALX-350-011 Enzo, 200 μM PMSF #6367.1 Carl Roth, 20 μM cOmplete #4693116000 Merck) on ice. Samples were centrifuged (13000 rpm, 4 °C) and pellets were discarded. Total protein amount in the supernatant was quantified by spectrophotometric Bradford Assay with Roti-Quant®. Samples were boiled in *Laemmli* buffer at 95 °C. Sodium-Dodecylsulfate-Polyacrylamide-Gel-Electrophoresis (SDS-PAGE) was used to separate proteins on 10 % acrylamide gels followed by Western Blot using the MiniProtean system (BioRad). Unspecific binding sites were blocked with Roti-Block ®and primary antibodies (Table 2) incubated overnight (1:1000) at 4 °C. Membranes were incubated with secondary antibodies (1.15 000) labeled with IRDye® (Table 3) and scanned at an Odyssey (LI-COR).

### 4.11 Immunoprecipitation

Biotinylated proteins from *BioID* experiments were pulled down with MyOne Streptavidin C1 Dynabeads™ (#65001 Invitrogen) according to manufacturers’ protocol. Co-IP of target proteins was performed similarly with the Dynabeads™Protein G IP Kit (#10007D Invitrogen). Briefly, cells were harvested and lysed like for Western blot. Beads were pre-cleared and 500 μg sample was used as input. Proteins were pulled down overnight by target antibodies (or IgG control) and incubated with beads (both overnight at 4 °C). Supernatant served as post-IP control. Target proteins were eluted from the beads and all amples were boiled in *Laemmli* buffer at 95 °C.

### 4.12 Scratch-wound assay

Physiological assays were performed with the IncuCyte® (Sartorius) system.

Scratch-wound assessment was carried out on 96 well image lock plates with 20 000 cells. After 1 day of growth in full medium, the Essen Wound Maker was used to set a defined scratch in each well. Media was exchanged to MEM +2 % FCS and optionally 5 ng/ml or 50 ng/ml human EGF (#AF-100-15 PeproTech). Migration was monitored for 4 days and analyzed with the IncuCyte S3 2021 software.

### 4.13 Statistical analysis

Data are presented as mean and standard error of the mean (SEM). All experiments were at least conducted in three independent biological replicates, defined by “n”. Calculations and statistical analysis were performed with Prism 10 (Graph Pad). p- Values smaller than 0.05 were accepted as statistical significant. ∗p < 0.05, ∗∗p < 0.01, ∗∗∗p < 0.001. In case of multiple statistical tests, Tukey *post hoc* correction was applied. Normalizations are indicated in the graphs.

## CRediT authorship contribution statement

**Dana Maureen Hebchen:** Data curation, Formal analysis, Writing – original draft. **Tim Schader:** Data curation, Formal analysis, Methodology. **Manuela Spaeth:** Data curation, Methodology. **Niklas Müller:** Data curation, Methodology. **Johannes Graumann:** Data curation, Methodology. **Katrin Schröder:** Formal analysis, Project administration, Supervision, Writing – review & editing, Conceptualization.

## Declaration of competing interest

The authors declare that they have no known competing financial interests or personal relationships that could have appeared to influence the work reported in this paper.

## Figures and Tables

**Table:** 

	Bovine Serum Albumin
	Colonadenocarcinoma 2 cells
	Cycloheximide
	Chloroquine
EEA1	Early Endosomal Antigen 1
EGF(R)	Epidermal Growth Factor (Receptor)
Erbin	Erbb Interacting Protein
Hek293	Human Embryonic Kidney cells 293
HUVEC	Human Umbilical Vein Endothelial Cells
IF	Immunofluorescence
IP	Immunoprecipitation
KO	Knockout
	Lysosomal-associated membrane protein 1
MCF7	Michigan Cancer Foundation 7 cells
MS	Mass spectrometry
NoxO1	NADPH oxidase organizer 1
	Overall Survival
	Protease Inhibitor Mix
PLA	Proximity Ligation Assay
	Primaquine
	Ras-related protein Rab-7,-11
	Relapse Free Survival
ROS	Reactive Oxygen Species
	Transcription Factor EB
WB	Western Blot
