# Supplementary material for: NoxO1 regulates EGFR signaling by its interaction with Erbin
Source: Redox Biol. 2024 Oct 16;77:103396. doi: 10.1016/j.redox.2024.103396 (PMC11536020; doi:10.1016/j.redox.2024.103396)
Supplement: Multimedia component 1 [file mmc1.pdf]

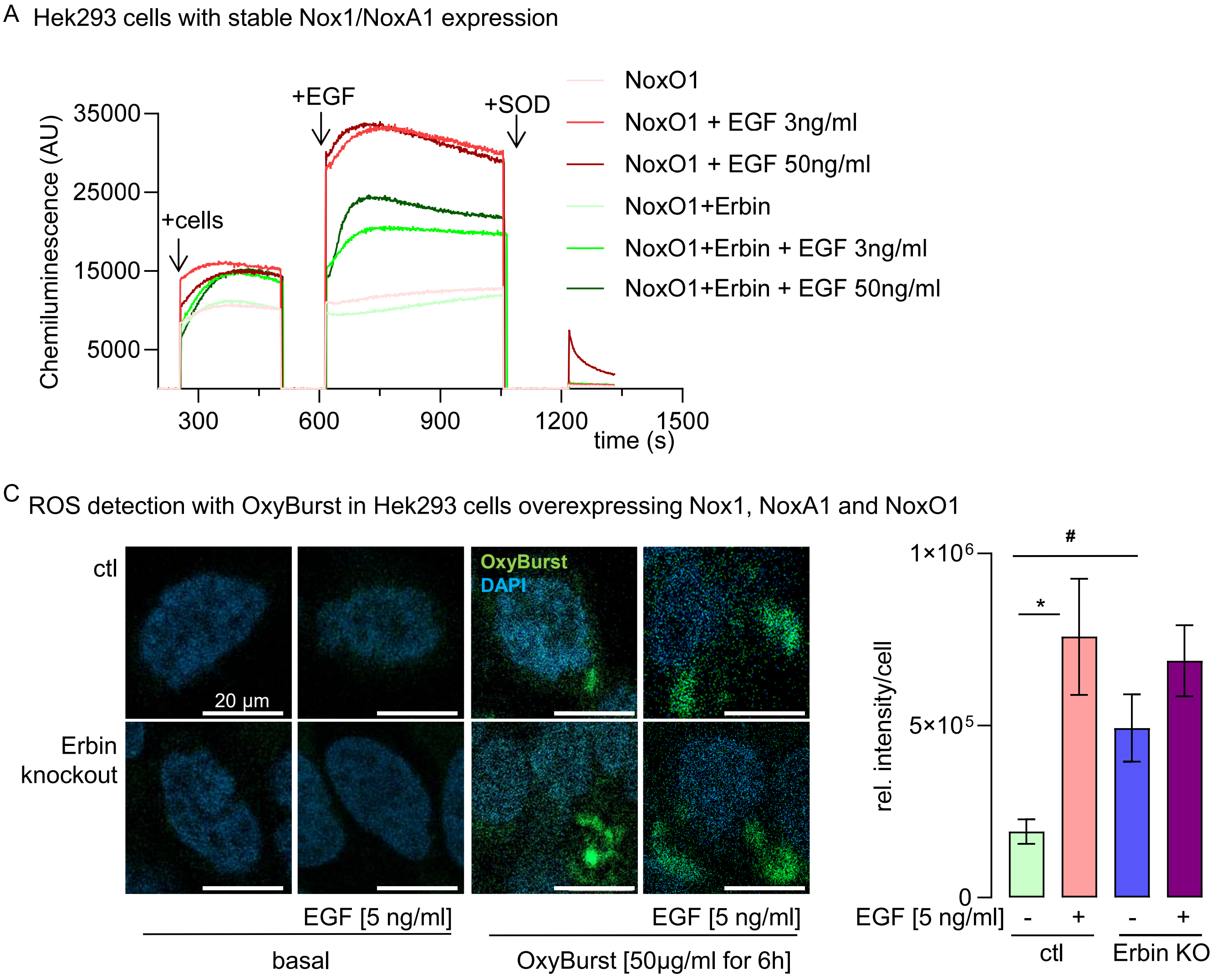

Supplemental Figure 1: EGF induced and NoxO1 mediated ROS formation depends on Erbin. Superoxide formation as measured in Nox1/NoxA1-Hek293 cells overexpressing NoxO1 with the aid of L-012. (A) ROS formation in Hek293 cells overexpressing without or in combination with Erbin (B) ROS formation in Hek293 cells loaded with the succinimidyl ester of OxyBURST Green H2DCFDA and treated with or without EGF for 15 min as indicated. n= 5; *p<0.05 ctl vs. EGF, #p<0.05 ctl vs. Erbin KO in Two-Way ANOVA + Tukey post hoc test; mean ± SEM;
